# Nutritional Strategies for Preterm Neonates and Preterm Neonates Undergoing Surgery: New Insights for Practice and Wrong Beliefs to Uproot

**DOI:** 10.3390/nu16111719

**Published:** 2024-05-31

**Authors:** Domenico Umberto De Rose, Alexandre Lapillonne, Silvia Iacobelli, Irma Capolupo, Andrea Dotta, Guglielmo Salvatori

**Affiliations:** 1 Neonatal Intensive Care Unit, “Bambino Gesù” Children’s Hospital IRCCS, 00165 Rome, Italy; irma.capolupo@opbg.net (I.C.); andrea.dotta@opbg.net (A.D.); guglielmo.salvatori@opbg.net (G.S.); 2 PhD Course in Microbiology, Immunology, Infectious Diseases, and Transplants (MIMIT), Faculty of Medicine and Surgery, “Tor Vergata” University of Rome, 00133 Rome, Italy; 3 Department of Neonatology, APHP, Necker-Enfants Malades University Hospital, EHU 7328 Paris Cite University Paris, 75015 Paris, France; alexandre.lapillonne@aphp.fr; 4 Children’s Nutrition Research Center, Baylor College of Medicine, Houston, TX 77024, USA; 5 Réanimation Néonatale et Pédiatrique, Centre Hospitalier Universitaire Saint-Pierre, BP 350, 97448 Saint Pierre CEDEX, France; silvia.iacobelli@chu-reunion.fr; 6 Centre d’Études Périnatales de l’Océan Indien (UR 7388), Université de La Réunion, BP 350, 97448 Saint Pierre CEDEX, France; 7 Donor Human Milk Bank, “Bambino Gesù” Children’s Hospital IRCCS, 00165 Rome, Italy

**Keywords:** enteral feeding, parenteral nutrition, residual gastric volume, refeeding syndrome, newborn, nutrition, necrotizing enterocolitis

## Abstract

The nutrition of preterm infants remains contaminated by wrong beliefs that reflect inexactitudes and perpetuate old practices. In this narrative review, we report current evidence in preterm neonates and in preterm neonates undergoing surgery. Convictions that necrotizing enterocolitis is reduced by the delay in introducing enteral feeding, a slow advancement in enteral feeds, and the systematic control of residual gastric volumes, should be abandoned. On the contrary, these practices prolong the time to reach full enteral feeding. The length of parenteral nutrition should be as short as possible to reduce the infectious risk. Intrauterine growth restriction, hemodynamic and respiratory instability, and patent ductus arteriosus should be considered in advancing enteral feeds, but they must not translate into prolonged fasting, which can be equally dangerous. Clinicians should also keep in mind the risk of refeeding syndrome in case of high amino acid intake and inadequate electrolyte supply, closely monitoring them. Conversely, when preterm infants undergo surgery, nutritional strategies are still based on retrospective studies and opinions rather than on randomized controlled trials. Finally, this review also highlights how the use of adequately fortified human milk is strongly recommended, as it offers unique benefits for immune and gastrointestinal health and neurodevelopmental outcomes.

## 1. Introduction

With increasing evidence-based practices, expert opinions have gradually been replaced by a deeper comprehension of neonatal needs based on the principles of optimizing growth, neurodevelopment, and long-term health outcomes [1]. Premature infants, especially very premature infants, face particular challenges related to the immaturity of the central nervous system. In addition to neonatal morbidities (such as sepsis, patent ductus arteriosus, bronchopulmonary dysplasia, and cerebral hemorrhage), inadequate nutrition and reduced growth are now considered important risk factors for abnormal development and long-term neurobehavioral complications [2,3,4,5]. In this context, the main objective is to provide adequate nutrition to compensate for the immaturity of the organs, especially the digestive tract. Therefore, parenteral nutrition (PN) is often necessary initially [6]. The common opinion is that premature infants do not have the physiological maturity to rapidly tolerate enteral feeding. Therefore, introducing enteral feeding is often delayed, and the daily increase in enteral feeding is limited by a fear of feeding intolerance (FI) and necrotizing enterocolitis (NEC). This leads to long fasting periods, prolonged parenteral nutrition, and inadequate nutritional support [7].

Similarly, preterm newborns undergoing surgery have even more specific nutritional needs linked to their initial condition. Before and after surgery, parenteral nutrition is necessary to ensure complete nutritional intake and prevent complications during the pre- and post-operative periods [8]. There may be differences in the timing of enteral feeding, depending on the type of surgical repair and the infant’s response [9]. The adequate feeding of these infants remains a challenge as they often cannot tolerate enteral feeding and/or cannot metabolize nutrients properly due to surgical stress. They suffer from hemodynamic instability, preoperative complications, metabolic stress, and inflammation, which can delay nutritional support [10]. An individualized feeding strategy that considers the specific surgical needs and the healing process is, therefore, essential for optimal postoperative recovery [11]. The use of human milk is strongly recommended, as it offers unique benefits for immune and gastrointestinal health and neurodevelopmental outcomes [12].

**Figure 1:**
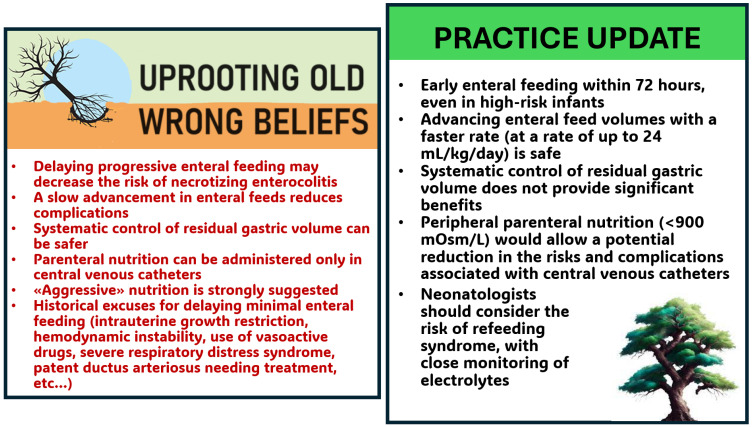
New nutritional strategies to put into practice and wrong beliefs to uproot.

Herein, we showcase current trends in feeding strategies for preterm infants, including those undergoing surgery, and recommend that clinicians should, where possible, apply evidence-based medicine into unit practice in order to deliver high-quality nutritional care (Figure 1).

## 2. Materials and Methods

In order to create this review, the terms “nutrition” and “neonate” were matched with “preterm”, “surgical”, or “surgery” in the PubMed database. All English-language papers that were recovered and released before 6 May 2024 were analyzed without imposing restrictions on date, country, study design, and outcomes, or inclusion/exclusion criteria. The reference lists of the identified papers were further checked, and each author identified additional references for this review based on their expertise in the relevant topic.

## 3. Standard Versus Individualized Parenteral Nutrition

In recent decades, efforts have been made to standardize PN for premature infants. The theoretical advantages are better physicochemical stability and aseptic production, especially if the standard PN (SPN) comes from large-scale industrial production [13,14], and a nutritional delivery that is less dependent on the prescriber’s knowledge. In this respect, SPN solutions can facilitate the early fulfillment of protein requirements in the first phase [15,16,17,18].

The European Working Group on pediatric parenteral nutrition conditionally recommended in 2018 that SPN solutions should generally be favored over individualized parenteral nutrition (IPN) solutions in most neonates, including very low-birthweight (VLBW) preterm infants, based on a low level of evidence (limited randomized controlled trials and some retrospective observational studies). Conversely, the combined working group recommended that IPN should typically be employed when the available range of SPN is insufficient to meet a patient’s nutritional needs (e.g., fluid and electrolyte expenses, surgery, and a prolonged requirement of parenteral nutrition) [19]. Indeed, IPN combined with pharmaceutical individualization may enhance protein intake, considering data from a randomized trial and an observational study [20,21]. IPN could provide greater daily glucose, amino acid, and lipid intake in VLBW neonates, as well as the ability to reach complete enteral feeding more quickly [21].

Overall, the choice of parenteral nutrition type (PN, SPN, or IPN) remains controversial, with the available studies providing very limited evidence [22]: SPN did not seem to lower rates of death, sepsis, or NEC, and PN duration compared to IPN [19,22]. To assess the true clinical benefit of SPN compared to IPN, sufficiently powered randomized controlled trials—RCTs—are needed, especially in lower gestational age cohorts and when surgery is needed.

## 4. Early Enteral Feeding and Fast Advancement in the Preterm Infant

It is preferable to begin enteral feeding as soon as possible [23]: it prevents villous atrophy, decreases intestinal permeability [24], and positively influences the intestinal microbiome [25].

Data from multiple observational studies indicate that adopting standardized enteral feeding procedures in the NICU enables preterm babies to get full enteral feeds more quickly. Short PN and hospital stays are permitted by unit guidelines to reduce NEC rates and perhaps enhance growth and neurological development [26]. Indeed, early enteral feeding has been demonstrated to lessen systemic inflammation and the risk of adverse outcomes (necrotizing enterocolitis, bronchopulmonary dysplasia, late-onset sepsis, and retinopathy of prematurity) [27]. A Cochrane meta-analysis, including 14 trials and 1551 infants, showed that delaying progressive enteral feeding in VLBW infants beyond four days after birth may not reduce the risk of NEC (relative risk—RR—0.81; low-certainty evidence) nor all-cause mortality (RR 0.97; low-certainty evidence). Delaying the introduction of enteral feeding may marginally lower the chance of developing FI (RR 0.81; low-certainty evidence) and most likely raises the likelihood of invasive infections (RR 1.44; moderate-certainty evidence) [28].

Overall, enteral feeding can usually be started within the first 72 h, even when conditions are severe, while low-to-moderate-risk preterm infants may benefit from its introduction in the first 24 h of life (Table 1) [29]. The rate of advancement that preterm infants can tolerate has been largely debated, and because of the fear of NEC, physicians have, until recently, tended to increase the amount of feeding slowly. However, this attitude may increase the risk of NEC rather than reduce it. For example, in a large nationwide study in France involving 3161 preterm infants, an increased risk of NEC was linked to slower and intermediate rates of enteral feeding progression strategies (adjusted odds ratios of 2.3 and 2.0, respectively) [30]. A recent Cochrane meta-analysis (14 trials for a total of 4033 infants) demonstrated that it is unlikely that the risk of NEC, FI, or death would be decreased by a gradual increase in enteral feeds up to 24 mL/kg/day as opposed to a quicker increase. Conversely, slowly increasing enteral feeds’ volumes could marginally raise the potential risk of invasive infection (RR 1.14; low-certainty evidence) [31]. Based on these findings, the most recent ESPGHAN position paper on enteral nutrition in preterm infants concluded that after 4 days of life, a faster increase in enteral feeding volume of 30 mL/kg/day) compared to a slower increase (15–20 mL/kg/day) does not significantly increase NEC or overall mortality rates [26]. Implementing standardized feeding protocols with these recommendations (such as the one we propose in Table 1), may help improve outcomes [26].

## 5. Early Enteral Feeding and Fast Advancement

The resumption of feeding preterm neonates after surgery is a minefield where the duration of fasting and the type of feeding can have both advantages and disadvantages. Clearly, the consequences of delayed enteral feeding include oral aversion, gastrointestinal effects (mucosal villous atrophy, dysbiosis, small bowel bacterial overgrowth, and intestinal dysmotility), dependence on parenteral nutrition, and intestinal failure-associated liver disease, as well as a higher risk of bloodstream infections due to the prolonged indwelling time of central venous catheters [32,33].

Premature infants with NEC, for example, are often treated with prolonged antibiotic administration and extended fasting for up to 14 to 21 days [34]. However, practices vary widely among centers, probably because the physiopathology of NEC is still poorly understood and because well-designed studies are scarce. As a result, while it is admitted that food intake should be suspended for a period of time depending on the condition’s severity, there are no clear recommendations on when food intake should be resumed once acute NEC has resolved. After medically treated NEC, there is no correlation between early enteral feeding resumed within 5–7 days after NEC diagnosis and a higher risk of unfavorable outcomes, including recurrent NEC or post-NEC strictures [35,36]. The cornerstones of post-NEC nutrition, therefore, include a careful assessment of the patient’s feeding tolerance, an individualized recognition of feeding tolerance, and a reduction in periods of intestinal rest and antibiotic exposure [32]. However, to date, there are no randomized controlled trials comparing different periods of bowel rest to determine the optimal duration. The practice recently suggested by several authors is to resume enteral feeding early (5–7 days) after the clinical and radiological signs of NEC have resolved [32,37].

In surgical NEC, the creation of a stoma is often necessary to allow adequate time for the inflamed gut to recover. There is very limited or even lacking evidence on the timing of the resumption of EN after surgical NEC [36]. A group of experts from the European Society for Pediatric Research recently summarized what is known about the nutritional management of surgically treated preterm infants with NEC [11]. Acute stress response brought on by critical conditions modifies the metabolism of macronutrients and is correlated with the length and severity of the damage. This critical illness, as it occurs in preterm infants with NEC, can be split into three phases: (a) acute phase, (b) intermediate phase, and (c) recovery phase [10]. Beyond the acute phase, modest enteral feeding could be resumed in the intermediate period, with regard to general conditions. In the recovery phase, which corresponds to the late postoperative phase when the inflammatory response has subsided or resolved, a gradual increase in enteral nutrition and a gradual cessation of parenteral nutrition have been proposed according to the improvement in intestinal adaptation.

Many infants with a stoma can achieve enteral autonomy, but this requires that the intestine above the stoma be sufficiently long. Infants with short bowel syndrome or with a jejunostomy often suffer from intestinal insufficiency and are dependent on parenteral nutrition for a longer period of time. In this case, specific guidelines should be followed [38]. In the advancement of enteral feeding following gastrointestinal surgery, the use of standardized guidelines appear to be safe and improve adherence to more evidence-based practices. Clinicians should consider the gestational age and birth weight, the length of residual bowel, the presence of a stoma and its location, the individual feeding tolerance, and other symptoms such as vomiting and abdominal distension [39,40].

Ostomy output should influence the advancement rate of enteral feeding: according to ESPGHAN guidelines on SBS, enteral nutrition can gradually be increased by 10–20 mL/kg/day if the stoma output is below 20–50 mL/kg/day or 6–10 stool/day; otherwise feeds should be reduced or withheld [41]. It is also critical to determine precisely which portions of the intestine are still functioning in a preterm infant with NEC who underwent surgery. The length and position of the surviving bowel and whether the ileocecal valve (ICV) is still intact are the three most important factors to ascertain [42]. This is because the ICV is a barrier that prevents the bacterial translocation of colonic contents and may slow down the transit time of the intestine [43]. In the case of resulting small bowel syndrome, signs of feeding tolerance include minimal emesis, a soft abdomen and appropriate abdominal X-ray, adequate stool and/or ostomy output (ideally <34 mL/kg/day), and adequate weight gain [42].

## 6. The Crucial Role of Human Milk

The advantages of human milk for preterm newborns have been shown by several research studies, even for those who underwent major surgery [44,45]. Human milk plays a crucial role in neurodevelopment through crucial nutrients, such as polyunsaturated fatty acids, that support brain development [46,47]. In recent years, attention has focused on the early introduction of human milk so that the newborn is no longer exposed to a long period of PN [48]. Expressed mother’s own milk (MOM), or, in its absence, donor human milk (DHM) through the use of milk banks, allows the provision of colostrum and human milk from the first days of life. This helps to strengthen the immune system, promote the development of the digestive system, and reduce complications [48,49]. In high-risk infants, such as those with a birth weight lower than 1500 g, DHM should be prioritized [50]. Indeed, there is moderate evidence about the advantages of DHM in preterm infants compared to formula [51].

For refeeding neonates after NEC, when human milk is unavailable, some neonatologists prescribe preterm formula, while others prescribe extensively hydrolyzed formula (EHF) [32]. If the gastrointestinal tract is severely damaged, elemental or lactose-free amino acid-based infant formulas with variable levels of medium-chain triglycerides can also be used due to malabsorption. However, to date, there are not enough research-based recommendations for optimal nutrition after NEC when human milk is unavailable [29].

## 7. The Importance of Fortification of Human Milk

In some cases, it is necessary to resort to fortification practices to ensure optimal nutritional intake, especially in cases of extremely premature newborns or those with special needs, such as surgical preterm newborns. A multidisciplinary discussion, including physicians, nurses, dietitians with training in pediatric nutrition, and developmental/feeding therapists, can represent the correct approach to optimize each nutrition care plan.

### 7.1. Available Fortifiers

Among the different options of fortifiers, a common practice is to use cow’s milk-based fortifiers. The last ESPGHAN position paper recommended using multicomponent fortifier solutions to boost the nutrient content of HM when birth weight is less than 1800 g [26].

An interesting alternative could be donkey’s milk as a basis for fortification, but the evidence is not strong enough to support the regular use of these products. Donkey’s milk is more similar to HM in composition than cow’s milk, with a protein and fat profile more suited to neonatal needs. Compared to a bovine counterpart in an isocaloric and isoproteic diet, the donkey milk-derived fortifier seemed to improve feeding tolerance with a similar auxological outcome in a recent trial [52]. Moreover, the availability of donkey’s milk is limited, making this option less viable on a large scale.

Recently, it has been proved that fortifying human milk (either MOM or DHM) with an exclusive human milk fortifier (EHMF) might be an intriguing possibility. This strategy has been linked to better early growth, as well as decreased death and morbidity. The initial adoption of an exclusive human milk diet (EHMD) and the exclusion of cow’s milk products may have long-term positive effects on health, according to preliminary research [53,54]. However, the availability of human milk-based fortifiers worldwide is still limited, and implementing EHMD programs meets several obstacles, regardless of the NICU size, patient population, or location [55].

### 7.2. Fortification of Human Milk in Preterm Infants

Even though HM is the best alternative for nutrition, the density of both macro- and micronutrients is inadequate to ensure optimum development, if it is not fortified. The goal is to raise the concentration of nutrients such that at the recommended feeding volumes (135–200 mL/kg/d), preterm infants receive quantities of all nutrients that fulfill their needs [56]. Two fortification methods could be adopted: (1) standard fortification, with an established quantity of fortifier added to a predetermined volume of HM; or (2) individualized fortification, intended as “adjustable fortification” (if protein amount is regulated according to blood urea nitrogen levels) or “targeted fortification” (when macronutrients are supplemented according to periodic human milk analysis) [57].

Pasteurized DHM should be used when the mother’s milk is unavailable for VLBW infants (<1500 g), but some NICUs also use DHM for preterm infants above 1500 g [58].

Recently, in a masked, randomized trial, extremely preterm infants ≤ 28 weeks fed maternal or donor milk were randomized to receive early (on feeding day 2) either a diet supplemented with an EHMF (intervention group) or a standard, unfortified diet (control group). Early fortification with a human-based product did not increase fat-free mass gain at 36 weeks but increased length growth velocity and reduced declines in head circumference-for-age Z-scores from birth to 36 weeks [54], which have been related to worse neurodevelopmental outcomes [4].

### 7.3. Fortification of Human Milk in Preterm Infants Undergoing Surgery

Currently, there is no evidence about HM fortification in preterm infants after gastrointestinal surgery, and the same indications for “non-surgical” preterm neonates should apply (<32 weeks and <1500–1800 g).

Considering that HM fortification increases milk osmolality, which has been linked to a greater risk of NEC, the osmolality of supplemented HM using multicomponent fortifiers (MCFs) and a protein fortifier (PF) was measured by Kreins et al. After fortification, osmolality rose dramatically, depending on the kind of fortifier that was utilized, and the maximum value was 484 mOsm/kg after 24 h [59].

Available data support the safety of current fortifiers in preterm infants in terms of necrotizing enterocolitis [60,61], and the supplementation with a human milk-based fortifier, as compared to a bovine milk-based fortifier, did not reduce the incidence of the composite outcome of NEC, sepsis, or death, or the incidence of different stages of NEC [62].

However, to date, there is no evidence of how to refeed preterm infants after NEC. A question postulated by Mayer and Kerner is the time of fortification in post-surgery feeding: the authors suggested that it is desirable to attain full-volume enteral meals before adding fortification to reduce variables [42].

Caution ought to be used in fortifying feeds in premature infants with serious congenital heart disorders (CHDs) since HM has a higher osmolality after fortification; this should be assessed before giving the milk to high-risk babies, if possible [63]. An unfortified EHMD may reduce the risk of pre-operative NEC in infants with complex CHDs [64]. After cardiac surgery, the timing of introducing fortified HM and which type of fortifier to use should be clarified with further studies [64], but the common concept is that HM fortification can be used to improve postoperative weight gain, reduce the hospital stay, and promote the infant’s early recovery after congenital cardiac surgery [64,65]. Until more evidence is available, it would be wise to proceed cautiously in order to hasten these patients’ growth, closely monitoring their tolerance [63].

## 8. The Role of Fetal Growth Restriction and Prenatal Doppler Anomalies

Clinicians should consider intrauterine growth retardation (IUGR) and the presence of antenatal comorbidities, such as abnormal umbilical arterial flow on Doppler in pregnancy. At birth, a targeted supply of nutrients is required to achieve optimal growth and avoid complications. Indeed, nutrient supply through the placenta is compromised in IUGR fetuses, leading to growth restriction [66]. Prenatal Doppler anomalies are linked to fetal hypoxia and may result in a redistribution of cardiac output, sparing the brain at the expense of splanchnic circulation and potentially predisposing it to gastrointestinal problems [67]. Poor outcomes are linked to the absence or reversal of end-diastolic flow (AREDF) in the umbilical artery, and elective preterm delivery is frequently performed: it is difficult to feed these babies, with some recovery during the first week of life. This justifies the gradual introduction of enteral feeding [68]. Indeed, infants born following AREDF in the umbilical artery and in the ductus venosus had significantly higher rates of FI, NEC, and spontaneous intestinal perforation (SIP), and achieved full enteral feeding (FEF) later compared to controls. An increased prevalence of FI and a longer time to reach FEF compared to controls were also observed among AREDF infants, in a context of brain-sparing redistribution, which likewise raised NEC rates. Assessing prenatal Doppler characteristics is thus crucial for estimating the individual risk of preterm newborns and identifying those who require individualized enteral feeding therapy [69].

This does not mean that these children should undergo prolonged fasting, considering that early feeding in IUGR-AREDF neonates permits earlier PN cessation and birthweight restoration and seems not to increase the incidence of FI and NEC [70].

## 9. Routine Monitoring of Gastric Residual for Prevention of NEC in Preterm Infants

Monitoring residual gastric volume in preterm newborns has become a topic of discussion in the clinical field, and many guidelines are progressively abandoning this practice [71,72]. Recent studies in this population have suggested that the systematic control of residual gastric volume does not provide significant benefits and may lead to unnecessary clinical interventions and interruption of enteral feeding [73,74,75,76]. In preterm newborns, gastric residual monitoring has traditionally been used to indicate feeding tolerance, but the current approach is moving towards a broader assessment of the newborn’s well-being through clinical signs and physiological parameters [71,72].

Concerning the possibility of refeeding versus discarding gastric residuals to speed up growth in preterm infants, giving partly digested milk, gastrointestinal enzymes, hormones, and trophic substances can help with digestion and increase gastrointestinal motility and maturation. However, aberrant residuals might cause vomiting, necrotizing enterocolitis, or sepsis. In a systematic review, Abiramalatha et al. found only limited data on the efficacy and safety of this practice from one small, unmasked trial (including 72 preterm infants). Refeeding gastric residual may have little to no impact on critical clinical outcomes (NEC, mortality prior to hospital discharge, the duration of enteral feedings, PN, and weight gain while hospitalized), according to low-certainty data [77].

The lack of evidence supporting the effectiveness of gastric residual monitoring, combined with an awareness of the risks associated with invasive procedures and the need to promote more evidence-based practices, is leading to a change in recommendations regarding this practice, underlining the importance of a complete clinical evaluation.

## 10. Peripheral Parenteral Nutrition

The possibility of administering low-osmolar parenteral nutrition (<850–900 mOsm/L) through peripheral venous access represents a further short-term strategy to follow, as soon as possible, in the newborn who already receives and tolerates a fair amount of enteral nutrition but who has not yet achieved complete enteral nutrition (full enteral feeding), such as those born after 30 weeks GA [78]. The use of low-osmolarity solutions can reduce the risk of complications, even if infused peripherally [78]. Potential complications of peripheral parenteral nutrition (PPN) include phlebitis, infiltration, or fluid overload issues. Frequent peripheral intravenous site rotations are required (usually every 48–72 h), and this should be considered. Administration through peripheral venous access also simplifies the management of the newborn patient to reduce the length of stay of the central venous catheters, which can lead to greater risks and complications such as infections and thrombosis [33,79,80].

A maximum osmolarity of 900 mOsm/L for PPN is usually suggested [80,81]. However, when comparing the incidence of line-related complications in patients receiving PPN with osmolarities of 1000–1250 versus <1000 mOsm/L, Fessler et al. could not find any significant differences [82].

## 11. Refeeding Syndrome in Preterm Infants

Refeeding syndrome (RS) is classically defined as a set of metabolic and electrolyte abnormalities brought on by the reintroduction and/or enhanced provision of calories following a time during which caloric intake was either missing or reduced [83]. In preterm newborns with IUGR, it is usually caused by inadequate transplacental nutrient transfer, followed by a relative excess of dietary availability, which is often supplied by parenteral nutrition [84], and is associated with increased morbidity and mortality. Among the biochemical indicators of refeeding syndrome in IUGR infants, hypophosphatemia is the signature of electrolyte derangement and alone may be already deemed enough for diagnosis [83]. Indeed, during prolonged periods of decreased or absent caloric intake, the body suppresses insulin secretion and relies on endogenous energy sources such as glycogen, amino acids, and fat. When carbohydrates are reintroduced, insulin secretion rises, and glucose becomes the primary fuel source. This shift increases the synthesis of phosphorylated glycolysis intermediates, given that phosphate is a vital component of adenosine triphosphate (ATP). Hypophosphatemia, a characteristic of RS, is caused by the increased demand for phosphorus in this anabolic state, low total body reserves (despite normal blood levels), and insulin’s activity to move phosphorus into the intracellular compartment [85]. The literature suggests defining hypophosphatemia as <4 mg/dL and severe hypophosphatemia as <2.5 mg/dL [86].

Furthermore, hypokalemia, hypercalcemia, and hypomagnesemia have been found in this inflammatory response. Hyperglycemia also occurs in RS [84].

Infants with hypophosphatemia alone or with only two laboratory abnormalities may be diagnosed with RS, as the likelihood of having three or more anomalies is less than 10% [87].

Severe hypophosphatemia may also occur in preterm infants (without IUGR), and it was for the first time described by Bonsante et al. as related to the incomplete provision of nutrients after the abrupt discontinuation of the placental nutrition at birth (Placental Incompletely Restored Feeding syndrome (PI-ReFeeding syndrome)) [88].

Indeed, this syndrome has become more apparent in preterm infants when neonatologists have moved toward aggressive early PN [86], with high intravenous amino acid intake in the presence of low electrolyte supply [10,89]. Closely monitoring electrolytes, especially phosphorus, is crucial in preterm infants [87]. In particular, optimizing phosphate and calcium intakes in intravenous nutrition solutions may reduce RS and its consequences [90].

## 12. The Relationship between Respiratory Distress Syndrome and Enteral Nutrition Tolerance

Respiratory distress syndrome and feeding intolerance are common conditions that are often associated in preterm neonates: an old conviction was that respiratory support can influence feeding tolerance, given that infants on non-invasive ventilation sometimes suffer from marked gaseous bowel distension [91].

Cresi et al. recently published the results of the ENTARES randomized clinical trial [92], in agreement with the previous retrospective results reported by Amendolia et al. [93]. They found that continuous positive airway pressure (NCPAP) and heated humidified high-flow nasal cannula (HHHFNC) had similar effects on feeding intolerance in 247 preterm infants <30 weeks GA. Clinicians may modify respiratory treatment by switching between the two non-invasive approaches based on efficacy and patient compliance without influencing feeding intolerance and no differences in the achievement of full enteral feeding [92].

Interestingly, Controzzi et al. proposed the use of lung ultrasound in supporting the clinical evaluation of feeding competence development in preterm neonates: the lung ultrasound score (LUS) may be used as a predictor of neonatal stability during deglutitory apnea as it is a sign of respiratory health. They showed in a small cohort of neonates < 32 weeks GA (n = 19) that the introduction of the first meal by bottle significantly correlated both with GA and LUS scores [94].

## 13. Higher Volume versus Standard-Volume Enteral Feeds

High-volume enteral feedings (>180 mL/kg/day) may enhance the growth and accretion of nutrients, which may improve neurodevelopment, compared to standard-volume feeds (up to 180 mL/kg/day) in preterm or very low-birthweight infants. High-volume meals, including fortified HM or preterm formula, enhanced weight growth throughout hospital stay (mean difference 2.58 g/kg/day; moderate-certainty evidence), according to a meta-analysis of data on 271 patients from two RCTs. One unmasked trial about unfortified HM or term formula reported that high-volume feeds improved weight gain during hospital stay (mean difference 6.2 g/kg/day; moderate-certainty evidence). However, the effects of these meals (≥180 mL/kg/day of fortified HM/preterm formula or ≥200 mL/kg/day of unfortified HM/term formula) on the risk of NEC and other outcomes are uncertain [95].

Moreover, high intake quantities should be used with care, particularly in babies with bronchopulmonary dysplasia (BPD) or a large patent ductus arteriosus (PDA) [95].

## 14. Antibiotics and Acid Suppressants Can Impact the Development of Gut Microbiome

Antibiotics are the most administered medications in NICUs worldwide because of early-onset and late-onset sepsis episodes [96]. However, in preterm, very low-birthweight infants, each day of antibiotic treatment has been associated with an increased risk of late-onset sepsis, NEC, or death [97]. Indeed, such exposure to early antibiotics can impact the developing gut microbiome [98], gastrointestinal transit [99], stool patterns, and oral feeding tolerance [100,101].

Similarly, treating feeding intolerance using acid-suppressive drugs is like a dog chasing its own tail. Ranitidine therapy has been associated with an increased risk of infections, NEC, and fatal outcomes in VLBW infants. Its use is not advised in neonatal age [102] and has been curbed by the drug’s availability: production was completely withdrawn by manufacturers in 2019 due to the presence of low levels of an impurity called N-nitroso-dimethylamine [103]. Parallelly, proton pump inhibitors have similar effects, creating dysbiosis of the microbiome in the mouth and gut [104], and a potentially higher incidence of infections and NEC [105].

Therefore, given that both these drug categories and the NICU stay can lead to intestinal dysbiosis [106], their use should be reduced in neonates [107].

## 15. New Monitoring Tools

Technology has revolutionized enteral nutrition monitoring in preterm infants, including those undergoing surgery. Advanced devices such as near-infrared spectroscopy (NIRS) provide neonatologists with real-time data, allowing the rapid adaptation of nutritional strategies in response to the newborn’s individual needs. NIRS provides a non-invasive monitoring of regional oxygen saturation (rSO2) [108].

A significant correlation has been reported between lower abdominal rSO2 values in the first week of life and the subsequent development of NEC [109].

NIRS can also be used to evaluate splanchnic oxygenation in response to enteral feeding and/or in response to blood transfusions, or it can indicate regional differences in comparison with cerebral oxygenation, which allows greater caution to be used in the advancement of enteral feeding [110,111,112,113,114,115].

Introducing enteral feeding in preterm newborns is sometimes complicated by the existence of a hemodynamically significant patent ductus arteriosus (PDA), which may impact mesenteric blood flow. NIRS can be useful for detecting a significant PDA [116]. The start of enteral feeding seemed not to appreciably alter the splanchnic oxygenation response to PDA, either with its restrictive or hemodynamically relevant aspects, and it was not linked to a higher incidence of gastrointestinal problems. This discovery might offer positive proof in favor of early enteral feeding for extremely preterm children with PDA [117].

To date, most studies were observational [109,118,119], and there is no evidence that NIRS-guided management could reduce the time to reach FEF and avoid complications in preterm infants.

## 16. Extrauterine Growth Restriction

Human growth is not constant through gestation and early infancy. Extrauterine growth restriction (EUGR) is a significant issue in recovering preterm newborns [120], given that those with EUGR based on weight, head circumference, or length are at risk of a poor neurodevelopmental outcome [121]. In particular, concerning weight, a growth velocity of about 20–30 g/kg/day is associated with infants’ maintaining or exceeding their birth weight Z-score [122]. Two main types of definitions of EUGR are now available in the literature:

1. “Cross-sectional” criteria, which delineate a threshold below which a newborn is deemed to be growth-restricted at a particular time point [120];

2. “Longitudinal” definitions, that assess the variation in growth between two time points, often starting from birth [123].

An expanding body of literature shows that a reduction in Z-score > 1 standard deviation in weight and head circumference, measured from when physiological weight loss ends and recognized as soon as possible instead of upon discharge, is a more reliable indicator of future neurodevelopmental outcomes in these children [4,124]. Thus, it is possible to talk about EUGR even before the 36th week or before discharge.

Daily weight changes are considered during the first week of life or during the period of postnatal weight loss in order to adjust fluid and electrolyte intakes. Afterward, it is necessary to abandon the “old” habit of simply considering weight gain from one day to the next, instead evaluating the growth trend over time. Growth in different parameters should be longitudinally monitored so that corrective measures may be implemented in a timely manner rather than after discharge [123].

## 17. New Frontiers: Artificial Intelligence and Nutrition

Artificial intelligence (AI) has the potential to significantly increase human understanding of illness and treatment effectiveness, even in neonatology [125].

Preterm infants’ intestinal perforation (IP) is a life-threatening disorder. It can occur spontaneously or as a result of necrotizing enterocolitis, requiring immediate surgical intervention (peritoneal drainage versus laparotomy) [126].

Predicting IP in babies with current protocols is tough, due to the multifactorial and complex nature of the disease [127]. AI can represent an early diagnostic tool for identifying neonates with IP. Machine learning (ML) models can be useful for meticulously assessing these difficult circumstances, starting with massive datasets of parameters and variables [125,128].

Irles et al., using a back-propagation neural network, identified some critical variables for IP prediction to pay particular attention to neonatal platelets and neutrophils, orotracheal intubation, birth weight, sex, arterial blood gas parameters (pCO_2_ and HCO_3_), gestational age, the use of fortifier, patent ductus arteriosus, maternal age, and maternal morbidity [129].

Concerning nutrition, Han et al. recently used AI to anticipate EUGR and, hence, organize timely nutritional strategies. ML models were created using various strategies on a large dataset (about 8000 extremely low-birthweight newborns from several Chinese units), and their feasibility and strong prediction performance were proved. However, the study’s usefulness was limited by the dataset’s lack of important information, even with its huge sample size [128].

Conversely, occasionally, the variation in DHM’s nutritional components (particularly in the first months after birth) makes it more difficult to produce a consistent pooled product and, as a result, to provide an adequate diet for VLBW infants. A fascinating study by Wong et al. revealed how ML algorithms can accurately estimate the macronutrient composition of DHM. The mean absolute error for the forecasts of individual donor and pooled fat was 0.91 g/dL and 0.42 g/dL, respectively. The macronutrient content of pooled milk had a lower prediction error, reinforcing the value of pooling practices [130]. Future studies should look at how milk bank pooling procedures can be facilitated by using forecasts of macronutrient content.

AI could also be used to identify variations in nutritional practices among different neonatal intensive care units: an example came from England, where agnostic, unsupervised machine learning was used to cluster a large clinical database on a large cohort of very preterm infants (n = 45,679) over a six-year period. The authors used two cases to demonstrate the possibility of discovering correlations between dietary practices and outcomes: discharge weight and BPD. They discovered the well-known impact of formula on increased weight at discharge, as well as evidence for the reasonable but weakly supported hypothesis that human milk protects against BPD [131].

## 18. Conclusions

The central role of nutrition is increasingly recognized, representing a decisive element for optimal brain growth, myelination, and neurological development. Enteral feeding should be introduced early and advanced without systematically controlling residual gastric volumes. Parenteral nutrition should be stopped as soon as possible, considering the option of an early removal of central lines and a peripheral infusion. Finally, the risk of refeeding syndrome should be considered, with the close monitoring of electrolytes. Even if recent research has allowed us to update these practices in preterm neonates without special needs, knowledge about factors influencing nutrition tolerance and adaptation in specific morbidities such as surgery remains sparse, with hypotheses and beliefs continuing to dominate the debate.

## Figures and Tables

**Table 1:** Proposed enteral feeding guidelines.

Birth Weight	Start Rate (mL/kg/Day)	How to Advance
≤1000 g	20 within the first 72 h after birth	Minimal enteral feeding for 3 days, then, if feeds are tolerated, +20
1001–1500 g	20	Minimal enteral feeding for 3 days, then, if feeds are tolerated, +30
>1500 g	30	Advance daily +30
>1500 g with congenital heart disorders *, prenatal Doppler abnormalities **, or cardiorespiratory instability	20 within the first 72 h after birth	Minimal enteral feeding for about 3 days, then, if feeds are tolerated, +10–20

* In preterm infants with congenital heart disorders (CHDs), enteral feeding should be started according to the pathology and other risk factors (surgical repair, prostaglandin infusion, etc.). ** Enteral feeding can be started after the first 24 *–48 h in preterm infants with prenatal Doppler abnormalities.*

## Data Availability

The original contributions presented in the study are included in the article, further inquiries can be directed to the corresponding author.
